# Evaluation of Platelet Lysate-Based Medium and Protein Substrate for HUVEC Culture and Expansion

**DOI:** 10.3390/biomedicines13051187

**Published:** 2025-05-13

**Authors:** Juan Manuel Duarte Rojas, Luz Marina Restrepo Múnera, Sergio Estrada Mira

**Affiliations:** 1 Tissue Engineering and Cellular Therapies Group–GITTC, Faculty of Medicine, University of Antioquia, Medellín 050010, Colombia; marina.restrepo@udea.edu.co (L.M.R.M.); sergio.estrada@udea.edu.co (S.E.M.); 2 Basic Biomedical Sciences Academic Corporation, University of Antioquia, Medellín 050010, Colombia; 3 Cellular Therapy and Biobank Laboratory, Hospital Alma Mater de Antioquia, University of Antioquia, Medellín 050010, Colombia

**Keywords:** human umbilical vein endothelial cells (HUVEC), human platelet lysate, platelet lysate serum, mixed medium, PL protein substrate, cell culture

## Abstract

**Background/Objectives:** Endothelial cell (EC) culture relies on specialized and commercial media with distinct growth supplement compositions. These media are expensive and must be imported, increasing the time to effective use. Human platelet lysate (PL) and platelet lysate serum (PLS) supplemented media are emerging alternatives to commercial media. **Methods**: Umbilical cords were collected, and human umbilical vein endothelial cells (HUVEC) were isolated and cultured using different media formulations, using Endothelial Cell Growth, Promocell^®^ (ECGM-Promocell^®^) commercial medium, and media supplemented with PL and PLS. **Results:** A mixed medium combining DMEM-F12 + PLS and ECGM-*Promocell^®^* maintained EC viability, adhesion, and proliferation. Introducing a PL-derived protein substrate enhanced cell adhesion and proliferation by simulating an extracellular matrix. Flow cytometry revealed positive CD31, CD144, and CD146 markers in cells cultured with ECGM-*Promocell^®^* and the mixed medium, with or without the PL-protein substrate. **Conclusions:** These findings suggest that the mixed medium, especially with the PL protein substrate, offers a cost-effective and efficient approach for EC culture and proliferation, holding promise for research and therapeutic applications.

## 1. Introduction

Endothelial cells (ECs) are a type of epithelial cells that line the inner surface of blood and lymphatic vessels, forming a continuous layer known as the vascular endothelium [1,2]. These cells are highly versatile, and their diverse functions are regulated by numerous factors present in the circulation or locally available in the vascular microenvironment. ECs express several surface antigens, such as CD31, CD34, CD144, CD146, and von Willebrand factor (vWF), which are used as markers to identify and characterize ECs in immunohistochemistry and flow cytometry studies [3,4,5].

The endothelium participates in several physiological processes, such as regulating vascular tone, establishing an immunological barrier, transmigration of leukocytes, blood coagulation, angiogenesis, and repairing damaged blood vessels. Endothelial function and dysfunction have been involved in inflammation, cardiovascular disease, long-term complications of diabetes, as well as tumor growth driven by exaggerated angiogenesis. Therefore, endothelial cell culture is crucial in vascular biology research [2,3,6]. Numerous scientific publications leverage endothelial cells in vitro, alone or in combination with other cell types, to study physiological and disease models, facilitating a better understanding of pathophysiology and guiding potential treatments [7].

After successful isolation, the expansion of ECs for subsequent experiments depends on the method’s effectiveness in achieving their proliferation and maintaining their viability. In vivo, endothelial cell proliferation is a highly regulated phenomenon, with vasculogenesis restricted to embryonic and placental development, while angiogenesis occurs in postnatal life to supply the demand for nutrients and oxygen as tissues grow.

In adulthood, this process is much more restricted, although it persists with other associated phenomena such as increased body fat, menstruation, or wound healing. This restriction causes the cell cycle of vascular endothelial cells to remain inactive in most of the vascular tree; however, during cell culture, endothelial cells are required to proliferate and expand for experimental or therapeutic use [1,3], which represents a significant challenge to researchers.

Human umbilical vein endothelial cells (HUVECs) are among the most widely used in human endothelial research and the development of potential cell therapies for various diseases, including cardiovascular, metabolic, neurological, and autoimmune diseases. Although this model does not represent all endothelial cell types found in the vascular tree, HUVECs have been used for their proliferative and differentiation capacity to study the properties of the vascular endothelium; in addition, it has been shown that these cells have an increased ability to secrete growth factors and cytokines that are important for tissue repair and regeneration [7,8,9].

To isolate endothelial cells, it is important to use appropriate culture media that provide essential nutrients and conditions for these cells’ growth, maintenance, and survival. The first culture media used for EC isolation and recovery were medium 199 (M199) supplemented mainly with different concentrations of fetal bovine serum (FBS), from 10% to 30%, as well as Eagle’s minimum essential medium (MEM) supplemented with FBS (8–10), and the MCDB131 basal nutritional medium, which is characterized by the presence of many components not found in previous basal media, including putrescine, adenine, thymidine, and higher levels of some amino acids and vitamins, as well as high levels of magnesium cofactor involved in cell proliferation. These additions make it possible to supplement the medium with low serum levels, but in most cases, it is still necessary to maintain supplementation with growth factors, hydrocortisone, and glutamine, among others [10,11].

Specialized and commercial culture media are supplemented with various growth factors to stimulate endothelial cell proliferation without affecting their phenotype or function. Indeed, optimizing culture conditions for EC proliferation is a recurring concern in vascular biology research [3]. Several endothelial cell culture media are available, which differing fundamentally in their composition of growth supplements [3].

While some media are supplemented with defined concentrations of recombinant growth factors, such as epidermal growth factor (EGF), fibroblast growth factor-2 (FGF-2), vascular endothelial growth factor (VEGF), and insulin-like growth factor-1 (IGF-1), others contain endothelial cell growth supplements derived from bovine brain extract (BBE), pituitary extract (BPE) or hypothalamus extract (BHE) and are rich in growth-promoting molecules of unspecified composition and amount. In addition to growth factors, endothelial cell media may be supplemented with hydrocortisone or may contain L-glutamine, heparin, ascorbic acid, and cyclic adenosine monophosphate (cAMP), as endothelial cell growth, proliferation, viability, and differentiation are regulated by endocrine factors [3,12].

Commonly used commercial culture media include endothelial cell growth basal medium (EBM, Lonza^®^; Basel, Switzerland) [13,14], complete endothelial cell culture medium (ECM, Endothelial Cell Medium; ScienCell; Carisbad, CA, USA) [15,16], vascular cell basal medium (ATTC^®^; Manassas, VA, USA) [17], and endothelial cell growth medium for large, medium, and microvascular tissue vessels (ECGM, *PromoCell*^®^; Heidelberg, Germany) [12,17,18]. These media contain essential nutrients and growth factors for endothelial cells [3,8,17,18].

Although it is possible to obtain them in our country, these specialized media are expensive and must be imported, which increases the time required before effective use (commercialization, availability, shipping, and delivery). In addition, most of these media are supplemented with FBS, which may contain factors interfering with endothelial cell growth and, therefore, their elimination from the medium may improve cell isolation and growth. Furthermore, in the case of cell therapy applications, it is preferable to use xeno-free media to reduce the likelihood of immunogenic reactions and post-infusion complications of these cells [19,20,21].

Both specialized and conventional culture media used for the nutrition, maintenance, and differentiation of endothelial cells must be carefully selected to ensure that they do not affect the physical, functional, and phenotypic properties of these cells.

In this context, the development of a more accessible, higher-yield, and xeno-free HUVEC culture system may facilitate broader access to research with these cells under conditions closer to their biological niche. This could represent a scientifically and economically viable alternative, particularly in low-resource settings or for translational studies requiring high reproducibility and clinical compatibility.

The aim of this work was to develop a culture medium supplemented with platelet unit derivatives (platelet lysate-PL and platelet lysate serum-PLS) and to compare its performance for the isolation, proliferation, and maintenance of HUVEC cells with the commercial endothelial cell growth medium, *Promocell* (ECGM-*Promocell^®^*) for vascular tissue engineering applications. This approach aims to provide a xeno-free, efficient, and biologically relevant culture system that better mimics the native endothelial environment, while also offering a more accessible and cost-effective option for vascular tissue engineering research.

## 2. Materials and Methods

### 2.1. Obtaining and Preparation of PL and PLS

Endothelial cell culture using PL and PLS preparations was performed as described previously [22].

Platelets unsuitable for transfusion were donated by the Blood Bank of the School of Microbiology, University of Antioquia. Each unit (50–65 mL) was preserved with CPDA-1 anticoagulant and tested negative for HTLV-1, hepatitis B and C, HIV 1–2, syphilis, and Chagas disease. Units were stored at −20 °C until use. Three hundred O blood group, Rh+ and Rh− platelet units were divided into three batches of 100 units. After thawing at 2–8 °C for 24 h and warming at 37 °C for 1 h, 5 mL from each unit was combined in sterile 500 mL bottles to form pools, which were then stored again at −20 °C.

Each pool underwent two freeze–thaw cycles to induce lysis. The lysate was centrifuged, filtered, aliquoted, and stored at −20 °C. For PLS, coagulation was induced with calcium gluconate, and the resulting serum was collected and stored similarly.

### 2.2. Analysis of Growth Factors and Cytokines in PL, PLS, and FBS Preparations

Evaluation of growth factors and cytokines in the endothelial cell growth medium (ECGM, *Promocell*^®^, Heidelberg, Germany) was performed as described previously for PL, PLS, and FBS [22].

### 2.3. Proliferation Assays of HUVECs with ECGM-Promocell^®^ Medium with and Without Protein Substrate

#### 2.3.1. Preparation of HUVEC Culture Dishes

Based on preliminary assays performed in the laboratory, low adherence and proliferation of HUVECs to the culture dish was observed (Supplementary Material, Figure S1); therefore, dishes pretreated with a protein substrate derived from PL were utilized. For its preparation, 0.0285 mL of PL/cm^2^ and 0.01 mL of calcium gluconate (B. Braund, Melsungen, Germany; 9.4 mg/mL; Calcium: 0.301 mg/mL) were added for each mL of PL, followed by incubation for 20 min at 37 °C with 5% carbon dioxide (CO_2_). HUVECs were seeded at 7.0 × 10^3^ cells/cm^2^. A control plate without the protein substrate was used. Photographic follow-up was performed at 4, 24, 48, 48, 72, and 96 h, and a count of the number of cells at various times was carried out. The assay was duplicated in three independent samples, with three images captured per well at each monitoring time. Image J software (2.9.0 version, Fiji) was used for image processing; regions of interest (ROIs) were manually selected. Thresholding was applied to segment structures. Area was measured to quantify size; shape descriptors assessed morphology; and pixel intensity was used to evaluate staining or signal levels.

##### Scanning Electron Microscopy Evaluation of HUVEC Culture Dishes with and Without Protein Substrate

Culture dishes were taken with and without the protein substrate and dishes with the protein substrate where the HUVEC were cultured. After cell culture, the samples were fixed with 10% buffered formaldehyde for 24 h. The formaldehyde was then removed, 2.5% glutaraldehyde was added, and the samples were left in this solution for 2 h. Successive dehydration steps were performed with 30%, 50%, 70%, 90%, 95% and 99% ethanol. Complete culture surfaces were sectioned, and the sections were left to dehydrate in a laminar flow chamber for 24 h. These were then fixed with graphite tape, coated with gold (Denton Vacuum Desk IV, Moorestown, NJ, USA), and evaluated in a scanning electron microscope (SEM) (JEOL JSM 6490 LV, Medellín, Colombia) under high vacuum conditions to obtain high-resolution images. The secondary electron detector was used to evaluate the morphology and topography of the samples.

#### 2.3.2. Isolation and Culture of HUVECs

Three umbilical cords were obtained from healthy pregnant mothers after their signing an informed consent form. Each cord was fragmented into approximately 10 cm sections in a laminar flow cabinet. The umbilical vein was cannulated and washed with antibiotic-containing saline (penicillin 500 U/mL and streptomycin 500 mg/mL; Lonza, Walkersville, MD, USA) to remove clots; then the ends of the umbilical vein were ligated, and collagenase type I at a concentration of 0.1% (1 mg/mL; Gibco, Life Technologies, Gran Island, NY, USA) was added to the interior of the umbilical vein, approximately 1 to 5 mL, depending on the caliber and length of the vein. It was incubated at 37 °C for 2 h, and manual pressure was applied along the umbilical vein to complete detaching of the endothelial cells. The extracted cell component was taken and centrifuged at 2500 RPM for 10 min to obtain the cell pellet. The supernatant was discarded, and the cells were resuspended in endothelial cell growth medium (ECGM, *Promocell*^®^) and seeded in pretreated or non-pretreated 25 cm^2^ culture dishes with the PL protein substrate for 10–15 days: upon completing ≥ 70% confluence. ECs were dissociated with 0.05% trypsin-EDTA (T/E) (Sigma-Aldrich, Darmstadt, Germany). Cell counting and viability were then performed using trypan blue.

#### 2.3.3. Evaluation of the Effect of PL, PLS, and FBS Concentrations on HUVEC Proliferation

Cell behavior in terms of proliferation and adhesion of HUVECs on PL substrate was assessed by comparing at 4, 24, 48, 72, and 96 h using different culture media, including DMEM-F12 supplemented with PL and PLS at concentrations of 5%, 10%, and 15%, and a standard medium (DMEM-F12 + 10% FBS).

#### 2.3.4. Evaluation of HUVEC Proliferation Using ECGM-Promocell^®^ Medium and Different Culture Media with PL and PLS

ECGM-*Promocell*^®^ medium, supplemented with 0.02 mL *v*/*v* FBS, 0.004 mL *v*/*v* ECGS supplement, 0.1 ng/mL human recombinant EGF, 1 ng/mL human recombinant FGF-b, 90 μg/mL heparin, and 1 μg/mL hydrocortisone, was compared with culture media supplemented with different concentrations (2, 4, 6, 8 and 10%) of PL and PLS, and with mixed ECGM-*Promocell*^®^-DMEM-F12 medium with ratios 10:90, 20:80, 30:70, 40:60, 50:50 and 60:40 supplemented with 9% PLS. HUVEC cell proliferation rate and cell behavior were evaluated at 24, 48, 72, 96, and 120 h.

#### 2.3.5. Evaluation of the Effect of PLS Concentration on HUVEC Proliferation

Based on the previous experiments, HUVEC behavior was evaluated using different concentrations of PLS between 5% and 50%. Adhesion and cell proliferation rates were determined at 24, 48, and 120 h to assess early adhesion, initial proliferation, and long-term cell growth. For the above assays, 100 U/mL penicillin and streptomycin (Lonza, Walkersville, MD USA), 2 μg/mL hydrocortisone (Vitalis, Bogotá, DC, Colombia), 1% L-glutamine (Lonza, Walkersville, MD, USA) were added to all culture media supplemented with PL and PLS, and 50 IU/mL sodium heparin (B. Braun, Melsungen, Germany) was added to media supplemented with PL.

All assays were performed in 12-well plates (3.5 cm^2^ per well), pretreated with PL protein substrate; HUVECs were seeded at 7.0 × 10^3^ cells/cm^2^. Each assay was conducted in duplicate for each medium and supplement concentration, and cell morphology, adhesion, and proliferation were compared by taking photographs in triplicate in each well. Image J software was used to analyze the results.

These experiments were designed to determine the optimal concentration of platelet-derived media for HUVEC proliferation and adhesion at different time points.

#### 2.3.6. Evaluation of the Effect of PL Substrate and Mixed Medium on HUVEC Phenotype

HUVECs were cultured in 25 cm^2^ dishes with and without PL protein substrate; cells were cultured with ECGM-*Promocell*^®^ medium and mixed medium (ECGM-*Promocell*^®^, DMEM-F12 with 9% PLS). The effect of protein substrate and medium on cell phenotype was evaluated. Phenotypic characterization was conducted by flow cytometry using the following antibodies: CD31 FITC (Clone: WM59), CD144 PE (Clone: 55-7H1), CD146 PE (Clone: P1H12) (Becton-Dickinson BD PharmingenTM, San Diego, CA, USA), CD34 FITC (Molecular Probes, Life Technologies, Waltham, MA, USA), HLA-DR FITC (Clone: L243) (Biolegend, San Diego, CA, USA). Samples analysis was performed at the Flow Cytometry Laboratory of the University of Antioquia’s Research Headquarters (BD LSRFortessa™ flow cytometer from Beckton-Dickinson). The results were analyzed using FlowJo™ software version 10.8.1 (BD, Biosciences, Franklin Lakes, NJ, USA).

### 2.4. Analysis of Results

All data are presented as mean ± standard deviation (SD). Statistical analysis of the data was performed using one-way ANOVA or two-way ANOVA, followed by Tukey’s multiple comparisons test, using GraphPad Prism software version 9.5.1 (La Jolla, CA, USA) with a significance level of *p* < 0.05.

## 3. Results

### 3.1. PL and PLS Present Higher Concentrations of Growth Factors and Cytokines Compared to FBS and ECGM-Promocell^®^

**Figure 1:**
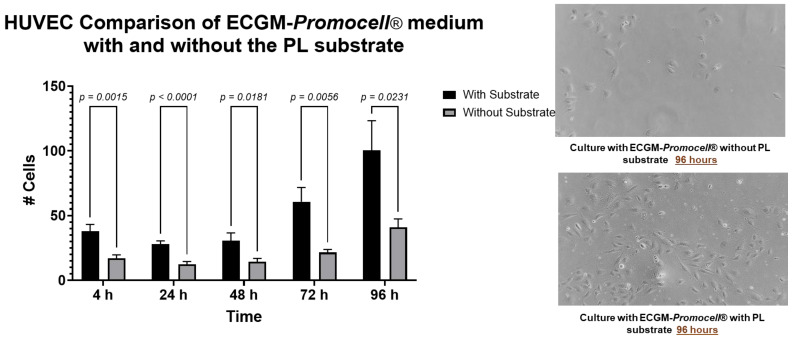
Comparison of ECGM-*Promocell*^®^ medium with and without PL protein substrate pretreatment in HUVEC culture. Data are shown as mean ± SD of cell number versus time (4, 24, 48, 48, 72, and 96 h). Evaluation of the proliferation kinetics of HUVECs in culture with ECGM-*Promocell*^®^ medium was carried out. At 96 h of culture, the number of cells is higher using the protein substrate. To determine statistical differences, two-way ANOVA was used, followed by Tukey’s test.

As described in [22], an analysis of growth factors in the different supplements and ECGM-*Promocell*^®^ demonstrated that FGF-b, TGF-B1, PDGF-AB, and IGF-1 were significantly increased in all PL and PLS preparations compared to FBS and ECGM-*Promocell*^®^ (*p* < 0.0001), as shown in Table 1. Additionally, EGF concentrations in PL preparations were higher concerning PLS, FBS, and ECGM-*Promocell*^®^ (*p* < 0.0001) (Figure 1).

The concentration of 9 cytokines and growth factors (IL-6, IL-10, RANTES, PDGF-AA, VEGF-A, TNF-α, IL1RA, GM-CSF, and G-CSF) involved in cell stimulation and differentiation, proinflammatory, and anti-inflammatory responses were evaluated by Luminex technique. As shown in Table 2, there were no significant differences in concentrations when comparing PL vs. PLS (*p* > 0.05). However, statistically significant differences were observed when comparing PL-PLS vs. FSB and PL-PLS vs. ECGM-*Promocell*^®^ in the concentration of all the molecules (*p* < 0.0005 PL-PLS vs. FBS; *p* < 0.0001 PL-PLS vs. ECGM-*Promocell*^®^).

### 3.2. The Use of a PL-Derived Protein Substrate Promotes Adhesion and Proliferation of HUVECs

Considering the low adherence of HUVECs observed in previous assays with conventional culture systems and ECGM-*Promocell*^®^ medium (Supplementary Figure S1), a PL-derived protein substrate was implemented to pretreat the culture dishes to be used in the HUVEC assays.

As shown in Figure 1, the HUVECs culture on a PL protein substrate improves cell adhesion and proliferation at all time points evaluated, being more relevant at 96 h of culture with ECGM-*Promocell*^®^ medium + pretreatment compared to cells seeded in this medium without dishes pretreatment (*p* < 0.005).

### 3.3. The Use of Protein Substrate Generates a Basis for HUVEC Growth and Adhesion

**Figure 2:**
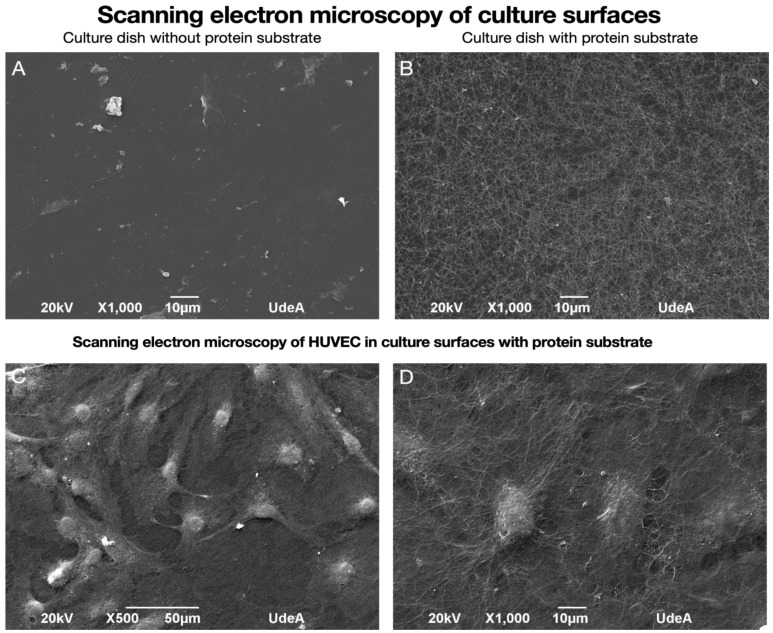
Scanning electron microscopy evaluation of culture dish surfaces and HUVEC adhesion. Images show the culture surface without the PL protein substrate (**A**) and the culture surface with the PL protein substrate (**B**). HUVECs are observed to adhere to the protein substrate (**C**,**D**), maintaining their morphological characteristics and normal proliferation.

As shown in Figure 2B, the use of the PL protein substrate on the culture surface resulted in the formation of a characteristic reticular mesh, typical of fibrin polymerization. This mesh simulates a structural scaffold resembling the extracellular matrix, which can support cell adhesion and function. In Figure 2C,D, HUVEC adhesion to the PL substrate is clearly observed, with cells firmly attached and spreading over the network, compared to the culture surface without this substrate (Figure 2A).

### 3.4. FBS Standard Concentration in Culture Media Does Not Promote HUVEC Proliferation

**Figure 3:**
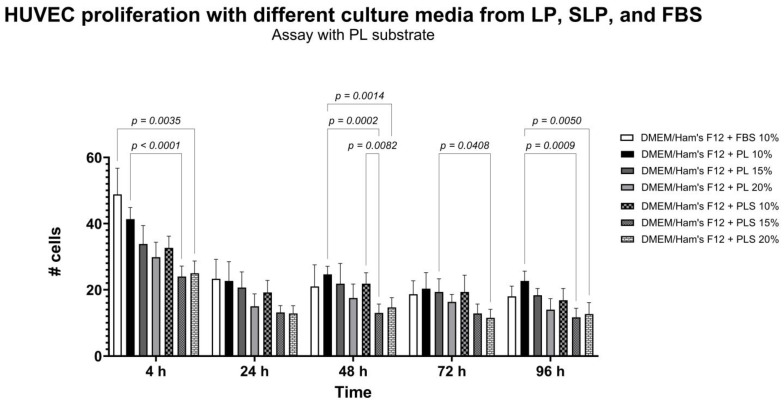
Comparison of different culture media with PL, PLS, and FBS with PL protein substrate pretreatment of dishes. Data are shown as mean ± SD of cell number vs. time (4, 24, 48, 48, 72, and 96 h). Evaluation of HUVEC proliferation kinetics was carried out using different media and concentrations of platelet derivatives and FBS. Normal expected proliferation is not observed; cells remain static over time. To determine statistical differences, two-way ANOVA was used, followed by Tukey’s test.

As shown in Figure 3, when evaluating HUVEC adhesion and proliferation using PL protein substrate and different culture media with PL and PLS concentrations between 5% and 20%, and with standard medium supplemented with 10% FBS, adequate cell proliferation kinetics were not observed, as cells remained static throughout the evaluation period.

### 3.5. HUVEC Culture with the Mixed Medium Is Similar Using ECGM-Promocell^®^ Medium

**Figure 4:**
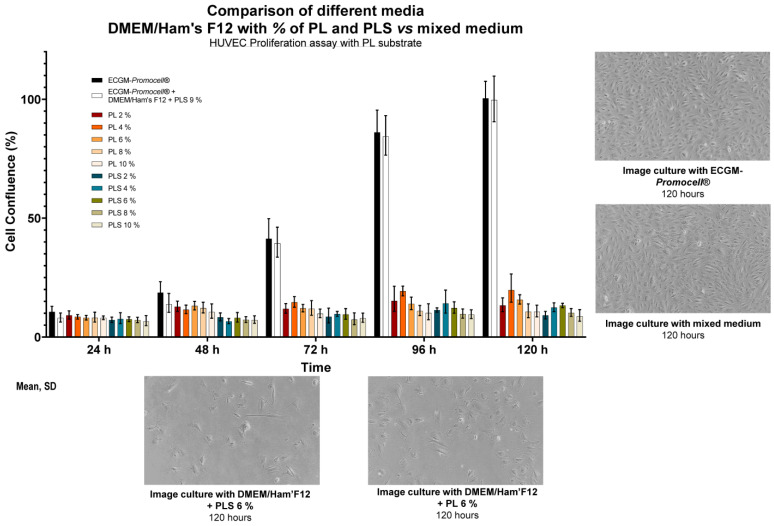
Comparison of the effect of different PL and PLS concentrations as DMEM-F12 supplements, mixed medium, and ECGM-*Promocell*^®^ on HUVEC culture and proliferation. Data are shown as mean ± SD of cell confluence percentage versus time (24, 48, 72, 96, and 120 h). Evaluation of HUVEC proliferation kinetics was carried out using different media and concentrations of platelet derivatives and comparing them with mixed medium and ECGM-*Promocell*^®^. Expected normal proliferation is not observed with DMEM-F12 media supplemented with different concentrations of platelet derivatives, in contrast with mixed medium and ECGM-*Promocell*^®^, where proliferation was normal and morphology was not affected, with cell confluence above 90% at 120 h.

As shown in Figure 4, when evaluating HUVEC adhesion and proliferation on PL protein substrate using culture media with concentrations of PL and PLS between 2% and 10%, compared to ECGM-*Promocell*^®^ and mixed medium, it was observed that cell expansion was not achieved in supplemented media with only platelet derivatives. The cells remained static during the follow-up time, with no observed differences even between PL and PLS.

**Figure 5:**
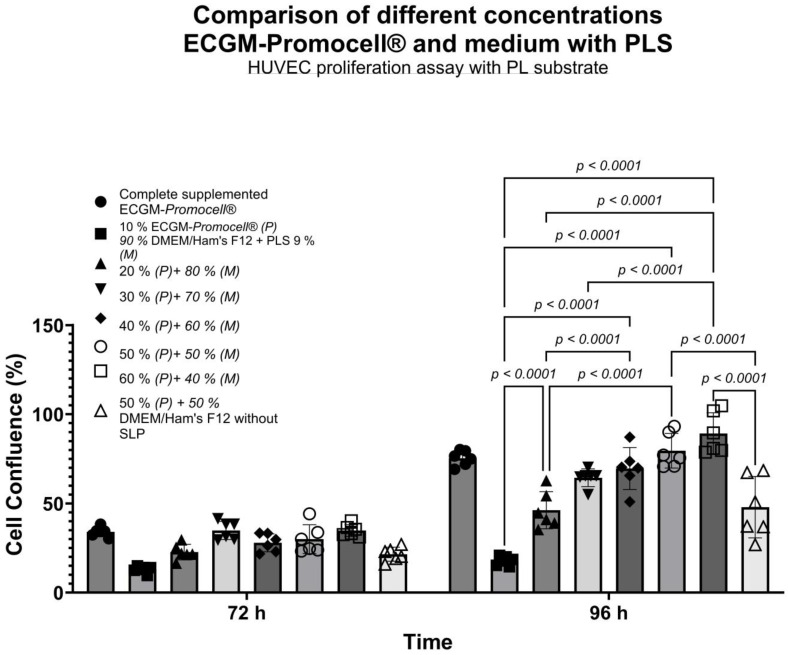
Comparison of different culture media with various combined proportions of ECGM-*Promocell*^®^ and mixed medium. Data are shown as mean ± SD of cell confluence percentage vs. time (72 and 96 h). Evaluation of HUVEC proliferation kinetics was carried out using different proportions of mixed medium and ECGM-*Promocell*^®^. A combination of ≤40% DMEM-F12 + 9% PLS and ≥50% ECGM-*Promocell*^®^ allows normal HUVEC proliferation kinetics. To determine statistical differences, two-way ANOVA was used, followed by Tukey’s test.

When HUVECs were cultured with the mixed medium (50% DMEM-F12 + 9% PLS and 50% ECGM-*Promocell*^®^), they showed similar behavior to ECGM-*Promocell*^®^ compared to other mixed media with different proportions (Figure 5), thus achieving a growth curve with a linear doubling rate up to 96 h (Figure 4 and Figure 5) with a confluence of 100% at 120 h (Figure 4).

### 3.6. Higher PLS Concentrations Do Not Enhance Cell Proliferation

**Figure 6:**
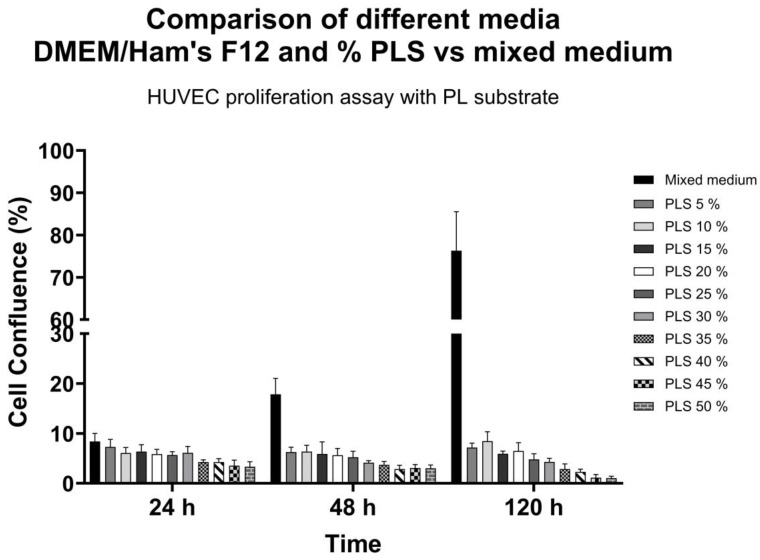
Comparison of DMEM-F12 with different PLS and mixed medium concentrations in HUVEC culture and proliferation. Data are shown as mean ± SD of cell confluence percentage vs. time (24, 48, and 120 h). Evaluation of HUVEC proliferation kinetics was carried out with different media and PLS concentrations and compared with the mixed medium. It was observed that the media with different PLS concentrations did not improve HUVEC cell proliferation compared to the mixed medium culture.

As shown in Figure 6, when HUVECs were cultured with higher PLS concentrations, a lower cell confluence percentage was observed concerning the culture with mixed medium, which could infer an inhibitory effect at high PLS concentrations.

### 3.7. Culture with PL Protein Substrate and Mixed Medium Does Not Affect the Phenotype and Morphology of HUVEC Cells

**Figure 7:**
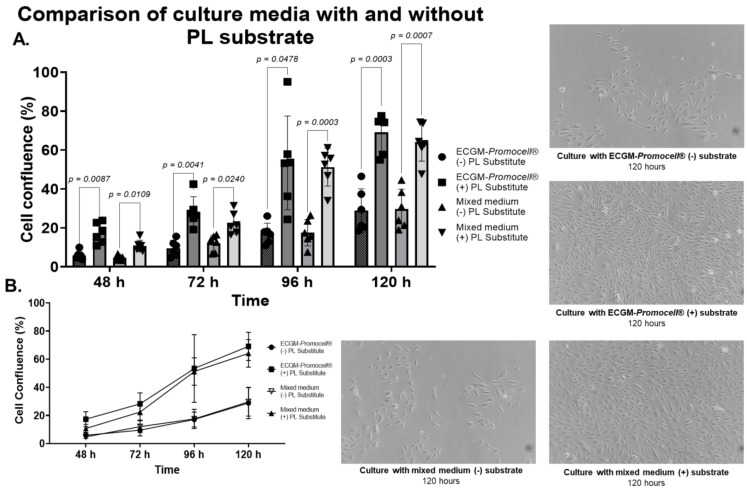
Comparison of mixed medium and ECGM-*Promocell*^®^ with and without protein substrate in HUVEC culture and proliferation. Data are represented in graphs (**A**,**B**) as mean ± SD of cell confluence percentage versus time (48, 72, 96, and 120 h). HUVEC proliferation kinetics were evaluated in culture with different media modifications (mixed medium and ECGM-*Promocell*^®^ with and without PL protein substrate). It was observed that mixed medium and ECGM-*Promocell*^®^ with PL protein substrate improved HUVEC adhesion and proliferation. To determine statistical differences in graph A, two-way ANOVA was used, followed by Tukey’s test.

**Figure 8:**
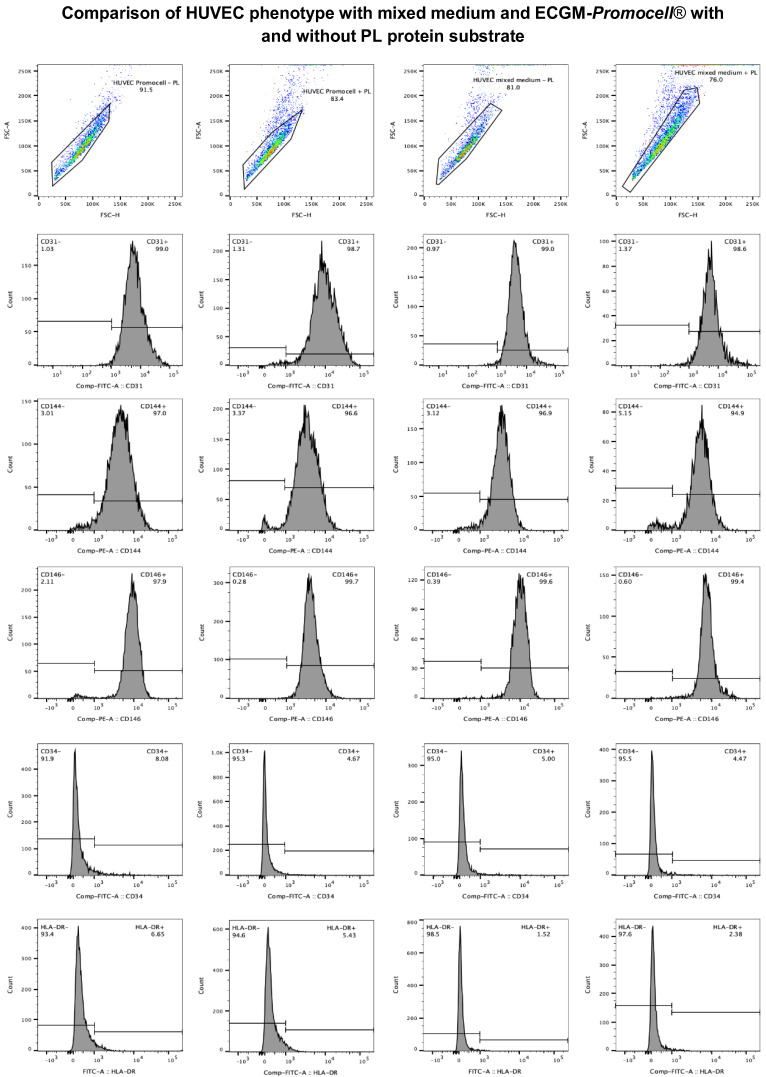
HUVEC phenotype with mixed medium and ECGM-*Promocell*^®^ with and without PL protein substrate. Data are represented in the graph as the expression level of surface markers CD31, CD144, CD146, CD34, and HLA-DR. Evaluation of the HUVEC phenotype was carried out in cultures with different media modifications (mixed medium and ECGM-*Promocell*^®^ with and without PL protein substrate). It was observed that CD31, CD144, and CD146 markers are expressed in more than 90%, while CD34 and HLA-DR markers are expressed in less than 10%. These results confirm that the profile of endothelial cells remains unaffected by any medium modification used.

Upon performing proliferation and morphology analysis, no significant differences were observed between ECGM-*Promocell*^®^ and the mixed medium (Figure 7). However, differences were observed between cultures with and without substrate. As previously demonstrated (Figure 1), the substrate significantly enhances HUVEC proliferation. Phenotypic characterization of HUVECs through flow cytometry revealed their expression of endothelial cell markers CD31, CD144, and CD146, and were negative for the hematopoietic marker CD34 and HLA-DR. No differences in the expression of these markers were observed either between culture media or with the use of the PL-protein substrate (Figure 8).

## 4. Discussion

Human endothelial cells are known to be difficult to culture in vitro. In the past, attempts to culture these cells have been carried out using conventional media designed for the growth of fibroblast cells (DMEM, MEM), e.g., with serum or media for the culture of different cell types without proteins, lipids, and growth factors, without serum (M199), or media for the protein-free growth of cells adapted to permanent cell lines (RPMI 1640). However, these media are usually unsuitable for endothelial cell growth because they do not provide the nutrients and growth factors necessary for their survival [10].

In several studies carried out, conventional media used for EC culture have been supplemented with high concentrations of animal-derived serum (FBS) [7,8,10,23,24]; however, these are not sufficient to maintain EC growth and morphology [17], as further shown in our study, where high concentrations of PLS also failed to maintain and did not have the best effect on cell proliferation and adhesion.

Although the platelet derivatives used in our study showed high concentrations of biochemical components, hormones, growth factors, and cytokines, these did not sufficiently nourish HUVEC effectively. On the other hand, upon analyzing the biochemical parameters described in [22], we found high levels of immunoglobulin G (IgG), calcium (in the case of PLS), and fibrinogen (PL) in the platelet derivatives. However, this would not be the cause of the poor success in HUVEC growth in culture media with the platelet derivatives, since as evidenced in [22], WJ-MSCs, fibroblasts, and AdMSCs grew and were maintained in culture with PL and PLS-supplemented media, in contrast to that described by Burnouf et al., where high IgG levels affected cell growth and differentiation [19]. No improvement was achieved after inactivating PL and PLS at 56 °C for 40 min to reduce IgG concentrations (Supplementary Figure S2).

Therefore, the culture medium is a critical factor in maintaining the typical properties of ECs in vitro. These cells are highly sensitive to their environment; thus, the culture medium must provide essential nutrients and conditions for cell growth, maintenance, and survival. Different types of specialized (commercial) media for HUVEC culture have been developed. These come in different presentations, either in media that are supplemented with defined concentrations of growth factors, or media that are supplemented with extracts whose concentrations and composition of cell growth-promoting molecules are not described in the product. These may also be supplemented with hydrocortisone, L-glutamine, heparin, ascorbic acid, and cyclic adenosine monophosphate (cAMP), which may be involved in ECs proliferation, viability, and differentiation [3].

However, specialized media are costly and have long import times, resulting in limited availability and restraining progress in research involving these cells. In this study, different culture media were formulated with platelet derivatives (DMEM-F12 with different PL and PLS concentrations), comparing them with the conventional standard medium with animal-derived serum (DMEM-F12 with 10% FBS) (Figure 3) and commercial endothelial cell growth medium (ECGM-*Promocell*^®^). When HUVEC were cultured using PL, PLS, and FBS, the same outcome as that achieved with ECGM-*Promocell*^®^ was not obtained. However, a mixed medium was implemented that combines ECGM-*Promocell*^®^ commercial medium with DMEM-F12 + 9% PLS, with results like those of commercial medium in terms of HUVEC culture and proliferation, which suggests that this option is a viable and cost-effective alternative for replacement at least 50% of the commercial medium mentioned, thereby reducing the high cost of this medium in our country.

In a study developed with retinal microvascular endothelial cells (REC), they evaluated the capacity of these cells to grow in standard cultures with DMEM (5% FBS) and with ECGM-*Promocell*^®^ commercial medium, as well as in combinations of different media of both [12]. A significant decrease in cell proliferation index was observed with DMEM, accompanied by changes in mRNA expression and tight junction proteins levels, as well as alterations in the subcellular localization of essential EC proteins such as von Willebrand factor, VE-cadherin, and claudin-5. Also, monolayer cell density and metabolic activity of RECs were affected for culture in DMEM. Although these effects are not clearly understood when using DMEM and FBS in EC culture, the authors describe that it is possibly due to high IL-6 secretion during cellular stress, the effects of tumor necrosis factor-alpha on cell permeability, or unidentified components in FBS [12]. These results are concordant with ours due to the limited success of HUVECs when cultured with DMEM combined with FBS, PL, and PLS.

Although the exact composition of ECGM-*Promocell*^®^ is not known due to intellectual property reasons, Bush et al., assume that its composition is based on that of MCDB131 medium, a composite cell culture medium designed to meet the specific requirements of microvascular EC [3,10,12]. An important difference is the tenfold higher concentration of Mg^2+^ in the MCDB131 medium (i.e., 10 mM) compared with the DMEM medium, where a significant increase in microvascular EC growth response was observed at high Mg^2+^ concentrations [10,12]. The high presence of this cation could enhance microvascular EC adhesion to extracellular matrix proteins achieved with protein substrate on culture dishes, since Mg^2+^ is an essential component of integrins and their complexes [25,26]. However, so far, it is not understood how cells can adhere to plastic supports without any extracellular matrix and how magnesium would help in this process. For example, in some cases, cells could express cell adhesion molecules, such as integrins or lectins, on their surface that directly interact with plastic components or indirectly with cofactors like magnesium. Other cells could secrete proteins, like fibronectin or laminin, which adhere to the plastic and act as anchors for the cells, or the medium used generates an environment rich in these adherent proteins [27].

Nonetheless, the maintenance of typical EC characteristics with ECGM-*Promocell*^®^ medium in cell culture is based on the combination of various components rather than a specific ingredient; to grow and expand HUVECs, the presence of hormones and other growth factors are essential to maintain the cells in long-term cultures [17]. In our study, a HUVEC proliferation assay was performed in dishes coated with PL protein substrate, using ECGM-*Promocell*^®^ medium, removing each supplementary component. It was observed that the lack of FBS and EC growth supplement (ECGS) led to a significant decrease in HUVEC cell proliferation, in contrast to the lack of the other components where cells continued normal proliferation (Supplementary Figure S3).

With these results and according to the literature reviewed, the addition of a source of growth factors, proteins, and hormones, such as an EC growth supplement (ECGS), would be the key to implementing a suitable medium for endothelial cell culture and maintenance, and would also be effective in preserving the phenotype of these cells [12]. This is demonstrated in previous studies investigating the optimal conditions of four different media for the proliferation and functional maintenance of human corneal ECs [28]. They concluded that a single medium does not provide all the nutritional conditions required by ECs and that these media require more factors and supplements to adequately simulate the nutritional environment of these cells [17,28,29,30].

On the other hand, it was observed that when using a protein substrate derived from human platelet lysate during HUVEC culture, better results in terms of cell adhesion and proliferation were achieved compared to cultures without this substrate, where cells could not be maintained in culture for more than three passages [17]. These results are not comparable with other studies, which report that coating culture dishes with a protein substrate may not be required [27,31]. In our study, using the PL protein substrate, we maintained the culture above passage 10 without affecting cell morphology and phenotype. The results obtained are comparable to those obtained by several authors when using a gel or protein component from fibronectin, type I collagen, fibrin gels, and laminin in their culture protocols to facilitate endothelial cell adhesion and growth [7,8,9,29,32,33,34,35].

The success of implementing this PL substrate in our study, promoting HUVEC adhesion and proliferation, can be explained by several factors: (I) PL preparations obtained in the laboratory contain high concentrations of growth factors, such as PDGF, TGF-β1, FGF-b, IGF-1, VEGF, and EGF, with essential bioactive molecules and cytokines for cell adhesion and migration; (II) it provides a protein-rich extracellular matrix structure that is important for ECs anchoring and interaction, simulating the natural environment of cells in vascular tissue, facilitating their adhesion and expansion [8,36]; (III) HUVECs and other endothelial cells have receptors on their surface, such as β1 integrins [8,25,26], which bind to proteins present on the PL substrate. These cell-substrate interactions promote adhesion and activate intracellular signaling pathways that regulate cell proliferation and behavior.

It is important to highlight that HUVECs are known to exhibit intrinsic biological variability due to donor differences and vessel-specific phenotypes. This inherent heterogeneity may limit the generalizability of our findings to other endothelial cell types. Therefore, our study represents an initial step toward the development of more accessible and xeno-free endothelial culture systems, and future research comparing different endothelial cell sources, such as microvascular or arterial endothelial cells, will be essential to validate and expand the applicability of this culture strategy.

## 5. Conclusions

Therefore, implementing a culture system combining endothelial cell growth medium (ECGM-*Promocell*^®^) and DMEM-F12 (50–50% respectively) with 9% PLS on PL substrate demonstrated superiority over commercial medium when used without the PL substrate. This strategy allows for a reduction of up to 50% in the use of commercial medium. It decreases associated EC culture costs, in addition to allowing the long-term maintenance of these cells in culture (>10 passages). Therefore, this culture strategy represents a promising and cost-effective alternative for researching and producing endothelial cells in our country and for other groups worldwide interested in obtaining well-characterized, sufficient, and suitable endothelial cells for research and therapeutic purposes.

## Figures and Tables

**Table 1:** Comparison of growth factors content in platelet derivatives, FBS, and ECGM-*Promocell*^®^. Growth factors IGF-1, PDGF-AB, FGF-b, TGF-β1, and EGF were analyzed by ELISA technique. Data are shown as mean ± standard deviation (SD) concentration of each factor (pg/mL and ng/mL in the case of IGF-1), across three batches of PL, PLS, and FBS, as well as ECGM-*Promocell*^®^. Both PL and PLS show higher concentrations of growth factors compared to FBS and ECGM-*Promocell*^®^. To determine statistical differences, one-way ANOVA was used, followed by Tukey’s test (FBS vs. PL o PLS: *** *p* < 0.0001; PL vs. PLS: ^+++^ *p* < 0.0001; Promocell vs. PL o PLS: ^~~~^ *p* < 0.0001).

	Supplements			
	PL	PLS	FBS	Promocell
IGF-1 (ng/mL)	7.364 ± 1.863 ***^, ~~~^	7.548 ± 1.227 ***^, ~~~^	0.000 ± 0.0	0.000 ± 0.0
PDGF-AB (pg/mL)	1697 ± 284.2 ***^, ~~~^	1813 ± 574.3 ***^, ~~~^	0.000 ± 0.0	0.000 ± 0.0
b-FGF (pg/mL)	1552 ± 104.0 ***^, ~~~^	1450 ± 126.1 ***^, ~~~^	0.000 ± 0.0	0.000 ± 0.0
TGF-β1 (pg/mL)	3660 ± 485.7 ***^, ~~~^	3780 ± 545.6 ***^, ~~~^	34.05 ± 20.40	0.000 ± 0.0
EGF (pg/mL)	1448 ± 320.6 ***^, ~~~, +++^	660.0 ± 183.5 ***^, ~~~, +++^	0.000 ± 0.0	0.000 ± 0.0

**Table 2:** Comparison of growth factors and cytokine concentrations in platelet derivatives, FBS and ECGM-*Promocell*^®^. Growth factors PDGF-AA, VEGF-A, and cytokines G-CSF, GM-CSF, IL-10, IL-6, IL-1RA, RANTES, and TNF-α, were analyzed using the Luminex technique. Data are shown as mean ± SD concentration of each factor (pg/mL), across three batches of PL, PLS, and FBS, as well as ECGM-*Promocell*^®^. Both PL and PLS show higher concentrations of growth factors and cytokines compared to FBS and ECGM-*Promocell*^®^. To determine statistical differences, one-way ANOVA was used, followed by Tukey’s test (FBS vs. PL o PLS: *** *p* < 0.0001; ** *p* < 0.001; *Promocell* vs. PL o PLS: ^~~~^ *p* < 0.0001; ^~~^ *p* < 0.001).

	Supplements			
	PL	PLS	FBS	Promocell
PDGF-AA (pg/mL)	2468 ± 210.3 ***^, ~~~^	2483 ± 189.6 ***^, ~~~^	0.000 ± 0.0	4.93 ± 5.49
VEGF (pg/mL)	168 ± 26.3 ***^, ~~~^	164 ± 21.3 ***^, ~~~^	0.000 ± 0.0	8.67 ± 1.2
G-CSF (pg/mL)	18.6 ± 6.5 ***^, ~~^	12.9 ± 0.74 ***^, ~~^	0.000 ± 0.0	2.87 ± 0.1
GM-CSF (pg/mL)	2.4 ± 0.43 **^, ~~^	2.4 ± 0.51 **^, ~~^	0.000 ± 0.0	0.000 ± 0.0
IL-10 (pg/mL)	18.6 ± 5.86 **^, ~~^	22.3 ± 7.6 **^, ~~^	0.000 ± 0.0	0.000 ± 0.0
IL-6 (pg/mL)	48.5 ± 14.8 **^, ~~^	56.9 ± 13.5 **^, ~~^	0.000 ± 0.0	0.48 ± 0.3
IL-1RA (pg/mL)	25.2 ± 5.2 **^, ~~^	27.0 ± 6.8 **^, ~~^	0.000 ± 0.0	0.57 ± 0.1
TNF-α (pg/mL)	103.48 ± 2.05 **^, ~~^	96.25 ± 2.3 **^, ~~^	0.000 ± 0.0	0.000 ± 0.0
RANTES (pg/mL)	5537 ± 540.8 ***^, ~~~^	4500 ± 1461 ***^, ~~~^	0.000 ± 0.0	328.0 ± 348.4

## Data Availability

The data presented in this study are available on request from the corresponding author.

## Supplementary Materials

The following supporting information can be downloaded at https://www.mdpi.com/article/10.3390/biomedicines13051187/s1: Figure S1: Comparison of HUVEC cells behavior cultured in DMEM/Ham-F12 supplemented with different FBS, PL, and PLS concentrations. Data are shown as mean ± SD of cell confluence percentage versus time (4, 24, and 48 h). Evaluation of HUVEC culture and proliferation behavior was carried out using different culture media, including DMEM-F12 supplemented with platelet derivatives and FBS, to the commercial ECGM-*Promocell*^®^ medium. At 48 h of culture, the cell count did not increase, the expected normal proliferation curve was not seen, and cells began to detach from the culture dish. To determine statistical differences, two-way ANOVA was used, followed by Tukey’s test; Figure S2: Comparison of different media with various concentrations of inactivated PL and PLS on HUVEC culture and proliferation. Data are shown as mean ± SD of cell confluence percentage versus time (24, 48, 72, and 96 h). Evaluation of HUVEC culture behavior and proliferation was carried out with different culture media using DMEM-F12 supplemented with 2%, 4%, 6%, 8%, and 10% inactivated PL and PLS. Expected normal proliferation was not observed with inactivated platelet derivatives. To determine statistical differences, two-way ANOVA was used, followed by Tukey’s test; Figure S3: Comparison of different modifications of ECGM-*Promocell*^®^ medium on HUVEC culture, proliferation, and adhesion. Data are shown as mean ± SD of cell confluence percentage versus time (24, 48, 72, and 96 h). Evaluation of HUVECs culture and proliferation behavior was carried out using different ECGM-*Promocell*^®^ medium modifications, in which each complementary component of the medium (complete ECGM-*Promocell*^®^, ECGM-*Promocell*^®^ without ECGS, FCS, hydrocortisone, FGF and without EGF) was removed. It was observed that HUVEC proliferation at 96 h was significantly decreased when ECGS and FBS were not added. To determine statistical differences, two-way ANOVA was used, followed by Tukey’s test.

## Glossary

AdMSC	Adipose tissue-derived mesenchymal stem cells
DMEM	Dulbecco’s Modified Eagle Medium
EC	Endothelial cell
ECGM	Endothelial cell growth medium
EGF	Epidermal growth factor
FBS	Fetal bovine serum
FGF-b	Basic fibroblast growth factor
G-CSF	Granulocyte colony-stimulating factor
GM-CSF	Granulocyte-macrophage colony-stimulating factor
HUVEC	Human umbilical vein endothelial cells
IGF-1	Insulin growth factor 1
IL-6	Interleukin 6
IL-10	Interleukin 10
IL1RA	Interleukin-1 receptor antagonist
PDGF-AB	Platelet-derived growth factor isoform AB
PDGF-BB	Platelet-derived growth factor isoform BB
PL	Human platelet lysate
PLS	Human platelet lysate serum
RANTES	Chemokine regulating activation expressed and secreted by T lymphocytes.
TNF-α	Tumor necrosis factor-alpha
VEGF	Vascular endothelial growth factor
WJ-MSC	Wharton’s jelly-derived mesenchymal stem cells
