# Supplementary figures and images for: Evaluation of Platelet Lysate-Based Medium and Protein Substrate for HUVEC Culture and Expansion
Source: Biomedicines. 2025 May 13;13(5):1187. doi: 10.3390/biomedicines13051187 (PMC12109029; doi:10.3390/biomedicines13051187)

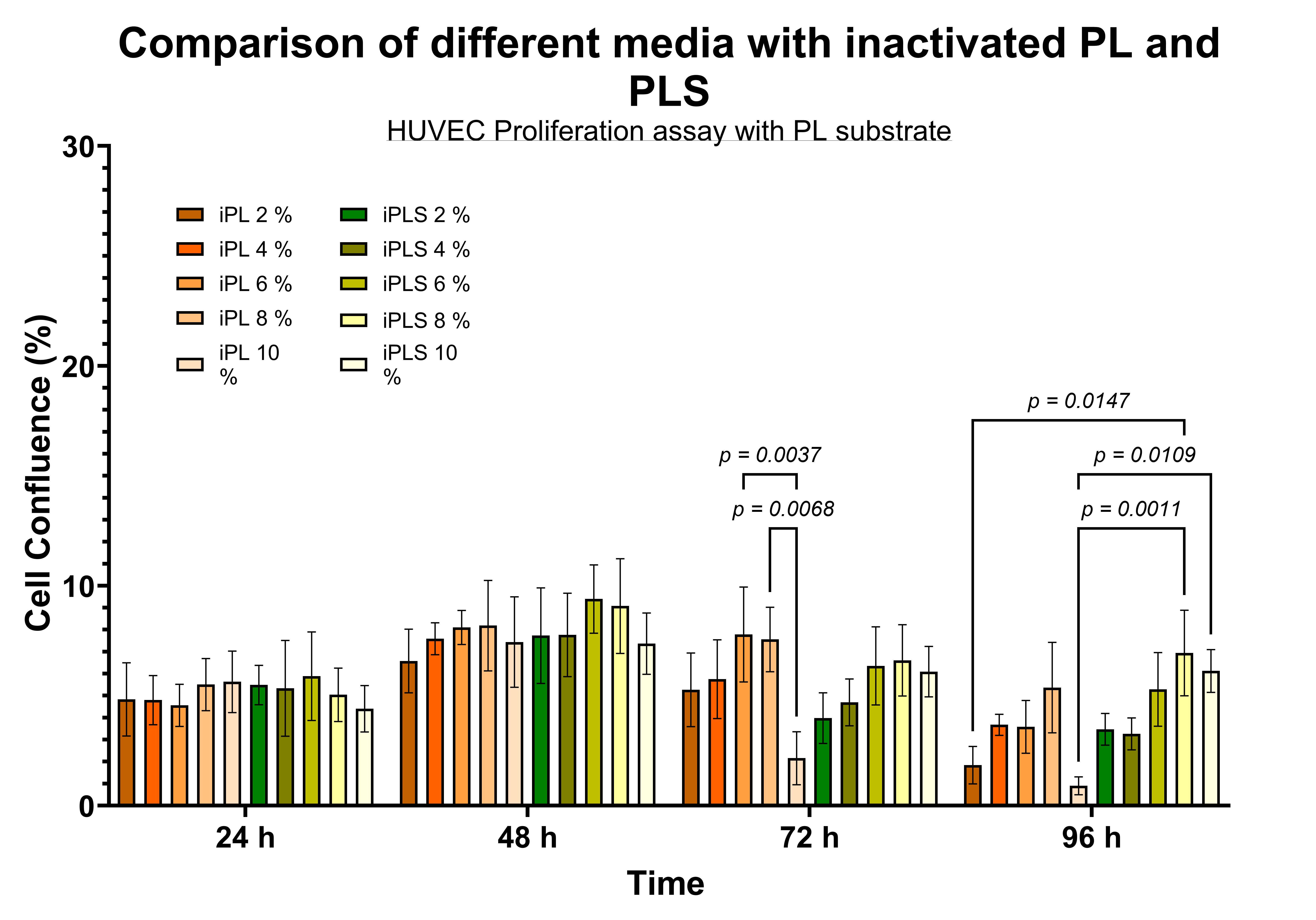

Supplement: Supplementary file 1 [file biomedicines-13-01187-s001.zip › Figure S2.jpg]
